# Nanocomposite Polymeric Materials Based on Eucalyptus Lignoboost^®^ Kraft Lignin for Liquid Sensing Applications

**DOI:** 10.3390/ma13071637

**Published:** 2020-04-02

**Authors:** Sónia S. Leça Gonçalves, Alisa Rudnitskaya, António J.M. Sales, Luís M. Cadillon Costa, Dmitry V. Evtuguin

**Affiliations:** 1 CICECO and Department of Chemistry, University of Aveiro, 3810-193 Aveiro, Portugal; leca.sofia@ua.pt; 2 CESAM and Department of Chemistry, University of Aveiro, 3810-193 Aveiro, Portugal; 3 I3N and Department of Physics, University of Aveiro, 3810-193 Aveiro, Portugal; jsales@ua.pt (A.J.M.S.); kady@ua.pt (L.M.C.C.)

**Keywords:** LignoBoost^®^ kraft lignin, potentiometric sensors, carbon nanotubes, impedance spectroscopy, transition metals

## Abstract

This study reports the synthesis of polyurethane–lignin copolymer blended with carbon multilayer nanotubes to be used in all-solid-state potentiometric chemical sensors. Known applicability of lignin-based polyurethanes doped with carbon nanotubes for chemical sensing was extended to eucalyptus LignoBoost^®^ kraft lignin containing increased amounts of polyphenolic groups from concomitant tannins that were expected to impart specificity and sensitivity to the sensing material. Synthesized polymers were characterized using FT-MIR spectroscopy, electrical impedance spectroscopy, scanning electron microscopy, thermogravimetric analysis, and differential scanning calorimetry and are used for manufacturing of all solid-state potentiometric sensors. Potentiometric sensor with LignoBoost^®^ kraft lignin-based polyurethane membrane displayed theoretical response and high selectivity to Cu (II) ions, as well as long-term stability.

## 1. Introduction

Conducting polymers comprise various types of polymeric materials with electronic and/or ionic conductivity—including doped conjugated polymers, redox polymers, polymer composites, and polymer electrolytes [1,2]. In the field of potentiometric sensors, conducting polymers are primarily used as ion-to-electron transducers for the fabrication of all-solid-state polymeric sensors. Conducting polymers can be employed as a solid inner contact or as a sensitive layer if it is mixed with active substances or contains functional groups capable of ion recognition. The merit of the latter approach is the possibility to diminish leaching of membrane components into aqueous phase, thus, increasing sensor lifetime and reproducibility while decreasing its detection limits [1].

Lignin is one of the main constituents of wood and is available as a waste product of pulp-and-paper industry. Technical lignins are highly branched irregular polymers that consist of phenyl propane units linked by a set of ether and carbon–carbon linkages and contain a variety of functional groups—such as hydroxyl, carbonyl, carboxyl, hydrosulfide, and sulphonate, among others [3]. These groups impart to lignin a capability to complex various compounds, including transition metals, pesticides and polycyclic aromatic hydrocarbons. Lignin also possesses redox activity that is attributed to the quinone structures formed during lignin oxidation serving as a reversible redox couple [4,5]. Both complexing and redox properties of lignin can be exploited in the chemical sensing and numerous applications of lignin as a sensing material in electrochemical sensors have been reported [6]. Impedimetric and amperometric sensors modified by Langmuir–Blodgett lignin films for the detection of copper, lead, cadmium, and humic substances were reported in [7,8]. Electronic tongues comprising lignin thin film sensors were applied to the recognition of taste substances and wines [9], and detection of triazine pesticides [10]. High electrocatalytic activity of oxidized lignin films allowed their use in electrode modification for the amperometric detection of ascorbic acid [4], nicotinamide adenine dinucleotide [5], and ozone [11]. Composites of lignin with nanoparticles and carbon nanotubes were used in electrode modification for the amperometric detection of trinitrotoluene [12] and chlorogenic acid [13].

As a method of sensing material preparation, covalent binding of lignin to the polymer matrix may be preferable to the thin film deposition as it would improve material stability and lifetime by preventing the leaching of low weight lignin fraction into the solution. Though lignin is insulating, its polyunsaturated nature promotes its transformation into conducting material upon appropriate doping. Synthesis of technical lignin-based polyurethanes, doped with carbon nanotubes for the sensing applications, has been previously reported [14,15,16]. It was demonstrated that doping with carbon nanotubes ensured electrical conductivity increase of kraft lignin-based polyurethane by 5 orders of magnitude [15]. Potentiometric sensors with lignin-based polyurethane membranes displayed selective response to Cr(VI) [16]. 

The purpose of this work was the synthesis of conducting lignin-based polyurethane and its application as a sensing material using a new type of lignin, LignoBoost^®^ kraft, as raw material. This lignin is isolated from black liquor after kraft cooking of eucalyptus wood by precipitation with CO_2_ within an advanced LignoBoost^®^ process. LignoBoost^®^ kraft lignin contains significantly more phenolic hydroxyl (almost double the amount due to the presence of associated tannins) and carbonyl groups compared to the conventional kraft lignin isolated from black liquor by acidification with mineral acid. Therefore, it was expected that the presence of a high amount of functional groups would impart to the sensing material based on Lignoobost^®^ kraft lignin polymer different sensitivity and selectivity properties. As this technical lignin will appear on the market, the evaluation of its applicability in specific areas is of particular importance. This study reports, for the first time, the conducting properties of LignoBoost^®^ lignin-based polyurethane and the potentiometric sensor manufactured therein as a sensitive layer. 

## 2. Materials and Methods

### 2.1. Reagents

LignoBoost^®^ lignin was produced at the pilot unit from the industrial black liquor after kraft pulping of eucalypt wood (*Eucalyptus globulus*) and supplied by The Navigator Company (Aveiro, Portugal). Poly(propylene glycol), tolylene 2,4-diisocyanate terminated with average Mn ~2300 (narrow MW distribution) with isocyanate content ~3.6 wt % (PPGDI), dibutyltin dilaurate, tris(hydroxymethyl)aminomethane (Tris) and aniline were from Sigma-Aldrich Química S.L. (Lisbon, Portugal). Multi-wall carbon nanotubes (MWCNTs) Nanocyl-3150 (purity > 95%, length 1–5 µm and diameter 5–19 nm) were from Nanocyl, S.A. (Sambreville, Belgium). Hydrochloric acid, sulfuric acid, potassium chromate; nitrates of calcium, cadmium, lead, zinc, copper, and chromium(III); and potassium hexacyanoferrate (II) and (III) were from Panreac Quimica S.A.U., Barcelona (Spain). Mercury(II) nitrate was from Fluka (Riedel-de Haën, Germany). All reagents were p.a. (for analysis) and acids were hyperpur-plus purity. Screen-printed electrodes (SPE) with carbon working, auxiliary electrodes and silver reference electrode were from DropSens (Oviedo, Spain). Ultrapure water (18 MΩ·cm^−1^) was used for the preparation of all solutions.

### 2.2. Polymer Synthesis and Sensor Manufacturing

Polycondensation reaction of lignin or ellagic acid with isocyanate was carried out using the procedure adapted from [16] and described in detail elsewhere [14]. Briefly, lignin or ellagic acid powder (250 mg), or their mixture with varying amounts of MWCNTs, was placed in the glass reactor with 1.5 mg of poly(propylene glycol), tolylene 2,4-diisocyanate terminated (PPGDI) and stirred for 40 min at 40 °C in order to obtain an homogenous viscous solution. Amounts of reagents were selected to ensure a ratio of 1.5 between isocyanate (NCO) groups in PPGDI and OH groups in lignin. The reaction was carried under nitrogen atmosphere. Concentration of MWCNTs were 0, 0.2, 0.4, 0.5, 0.8, 1.0, and 1.4% (w/w) of the polymer. After the homogenous mixture was obtained, the temperature was increased to 60 °C and the catalyst (dibutyltin dilaurate, ca 2%) was added. The mixture was stirred for further 10 min until it started to thicken. After that, it was removed from the reactor and used for the preparation of the sensor membranes and polymeric films. Films (about 1 mm thickness) used for the polymer characterization were prepared by pouring still liquid polymer into the flat mold. Polymeric films were cured during 4 h at 60 °C and sensors at room temperature for 24 h. Polymer of each composition was synthetized at least twice. 

Potentiometric sensors with and without solid inner contact were fabricated using SPE. Firstly, surface of SPE working electrode was rinsed with ethanol and water and cleaned by cycling potential for 5 cycles between −0.2 and +1.2 V at 5 0 mV·s^−1^ in 50 mmol·L^−1^ sulfuric acid. Solid contact was prepared by electropolymerization of aniline in deaerated aqueous solution of 50 mmol·L^−1^ aniline in 1 mol·L^−1^ hydrochloric acid by cycling potential for 100 cycles between −0.23 and +0.85 V at 50 mV·s^−1^. Sensors were washed with deionized water, conditioned for 2 h in 1 mmol·L^−1^ hydrochloric acid and dried. All controlled-potential experiments were performed with an EZstat-Pro EIS (NuVant Systems Inc., Crown Point, IN, USA). Platinum wire served as the counter electrode and Ag/AgCl (KCl 3 mol·L^−1^) served as a reference electrode. Sensors were prepared by placing a drop of liquid polymer on the working electrode of SPE. At least two sensors of the same composition were prepared.

### 2.3. Polymer Characterization

Lignin-based polyurethanes were characterized by Fourier transform mid-infrared spectroscopy (FT-MIR) using MATTSON 7300 series spectrometer (Mattson Instruments, Madison, WI, USA), equipped with total attenuated reflectance accessory SPECAC Golden Gate-Diamond, in the wavenumber range 4000–600 cm^−1^, with a resolution of 4 cm^−1^ and 128 scans per sample.

Differential scanning calorimetry (DSC) analysis was carried out using Perkin Elmer Diamond Differential Scanning Calorimeter (Waltham, MA, USA) with power compensation. Measurements were made under nitrogen flow of 40.00 mL·min^−1^ in the temperature range from −100 to 50 °C, with a heating rate of 10 °C·min^−1^ using approx. 10 mg of the sample placed in a platinum cap. 

Thermogravimetric analysis (TGA) was carried out using thermogravimetric analyzer SETSYS by Setaram Instrumentation (Caluire, France), equipped with vertical balance. Measurements were made under nitrogen atmosphere, in the temperature range from 20 to 800 °C with a heating rate of 10 °C·min^−1^ using approx. 8 mg of the sample placed in a platinum cap.

SEM images of lignin and lignin-based polyurethanes were recorded using Hitachi S-4100 microscope (Tokyo, Japan) on the carbon coated samples and applying acceleration voltage of 25 kV. SEM images of ellagic acid-based polyurethanes were recorded using bench TM4000Plus Hitachi microscope equipped with backscattered electrons (BSE) detector, using the uncoated samples and applying acceleration voltage of 15 kV.

DC electrical conductivity was measured at the temperatures between −110 and 100 °C using a 617 Keithley electrometer (Cleveland, OH, USA). Electrical contacts were made by painting polymer films on both sides with silver paste, simulating a parallel plate capacitor with a surface area of about 1 cm^2^ and 3 mm distance between electrodes.

Dielectric measurements for frequencies between 100 Hz and 1 MHz were carried out using an Agilent 4294A precision impedance analyzer at temperatures between −73 and 127 °C under helium atmosphere. Estimated relative errors on both, real and imaginary parts of the complex permittivity, were below 2%.

### 2.4. Potentiometric Measurements

Electrochemical measurements were carried out in the following galvanic cell:

Ag|AgCl, KClsat|sample|polymer membrane|carbon

Emf values were measured vs. Ag/AgCl reference electrode with precision of 0.1 mV using a custom-made multichannel voltmeter with high input impedance connected to the PC for data acquisition and processing. Calibration measurements were made in the solutions of zinc nitrate, cadmium, lead, copper, mercury, iron (III), and potassium chromate in the concentration range 10^−7^–10^−4^ mol·L^−1^. Tris with concentration of 1 mmol·L^−1^ and pH 7, adjusted by addition of hydrochloric acid, was used as supporting electrolyte. Redox response was studied in the solutions of two redox pairs, Cr (III)/Cr(VI) and Fe(CN)_6_^3−/4−^. Total concentration was 1 mM for both pairs, with the ratio of oxidized to reduced form varying from 0.01 to 100. Measurements in the solutions of chromium (III), iron (III), chromate and redox pairs were made at pH 2 on the background of 0.01 mol·L^−1^ HCl. Selectivity was estimated using mixed solution method. At least three replicated calibration measurements were run for each ion. Parameters of Nernst equation, i.e., slope of the electrode function and standard potential were calculated using linear regression and averaged over replicated calibration runs and sensor compositions.

## 3. Results

### 3.1. DC Conductivity 

Electrical conductivity is one of the requisites for the sensing material to be used as a membrane in potentiometric sensors. According to the previous work, lignin-based polyurethanes are insulating with conductivity of nearly 10^−8^ S·m^−1^, which can be increased several orders of magnitude by doping with carbon nanotubes [15,16]. Lignin is considered to be an excellent dispersant of carbon nanotubes in the polymeric matrix [14]. As phenolic hydroxyls are much less reactive to isocyanates than aliphatic ones [17], the redox properties of lignin involved in copolymerization may not change significantly. The advantage of this approach is a percolation effect observed in lignin-based polymers doped with MWCNTs, e.g., drastic increase of conductivity in the presence of small amounts of carbon nanotubes. Thus, conducting polymers can be obtained in a cost-effective manner and without changing other properties of the material. A similar approach was adopted in this work to increase the conductivity of the eucalyptus LignoBoost^®^ kraft lignin and ellagic acid-based polyurethanes. Ellagic acid was used in this study as the main eucalyptus kraft lignin contaminant of polyphenolic origin [18]. This tannin contributes substantially to the phenolic functionalities of the isolated lignin. 

**Figure 1:**
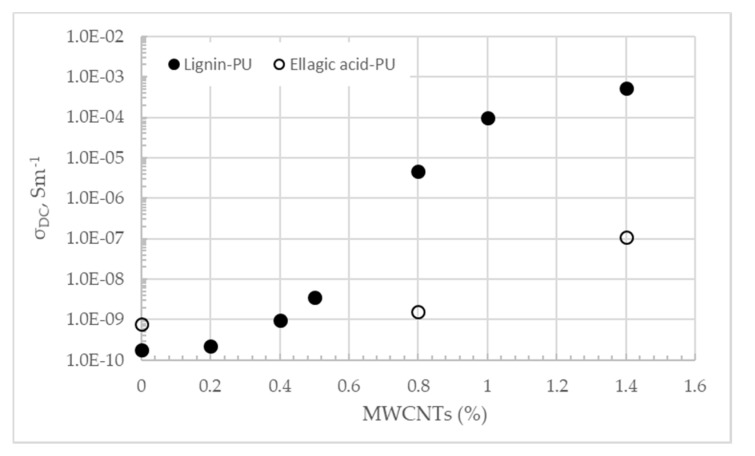
DC electrical conductivity σ_DC_ at constant temperature (293 K) as a function of the MWCNTs concentration of the LignoBoost^®^ kraft lignin and ellagic acid-based polyurethanes.

Firstly, polymers doped with 0.8 and 1.4% (w/w) of MWCNTs were synthesized. DC electrical conductivity (σ_DC_) of both polymers, as a function of the MWCNTs’ concentration at 293 K, is plotted in the Figure 1. The conductivity of both undoped polymers was very low: 1.80 × 10^−10^ and 7.68 × 10^−10^ S·m^−1^ for LignoBoost^®^ kraft lignin and ellagic acid-based polymers, respectively (Figure 1). Higher conductivity of polyurethane (PU) based on undoped ellagic acid, when compared to that of based on kraft-lignin, could be explained by the better dispersion of low molecular weight polyphenolic molecules in PPGDI copolymer than the larger lignin oligomers. Incompletely dissolved highly swollen aggregated lignin was previously detected in PU obtained by copolymerization of conventional acid-precipitated eucalyptus kraft lignin and PPGDI [14]. In addition, conjugated aromatic rings of flat ellagic acid structure could positively contribute to the conductivity of the final PU more than kraft lignin, whose structural units are predominantly linked by alkyl–alkyl, alkyl–aryl, and aryl–ether bonds, i.e., having less conjugated aromatic structures [18].

Addition of carbon nanotubes led to a significant increase in the conductivity of the LignoBoost^®^ kraft lignin-based polymer, reaching 5.37 × 10^−4^ S·m^−1^ after the addition of 1.4% (w/w) of MWCNTs. Despite the fact that conductivity of undoped ellagic acid-based polyurethane was higher compared to lignin-based one, the effect of the MWCNT addition on conductivity was less accentuated. The conductivity of only 1.12 × 10^−7^ S·m^−1^ was observed at 1.4% (w/w) of MWCNTs (Figure 1).

LignoBoost^®^ kraft lignin-based polymers doped with intermediate concentrations of MWCNTs were synthetized with the aim to confirm that conductivity of this polymer follows percolation scaling law and to determine percolation threshold (Figure 1). Percolation threshold x_c_ and critical exponent t were calculated by fitting experimental data to the Equation (1)

(1) $$\sigma_{DC}\approx {\left(x-x_{c}\right)}^{t}$$

where σ_DC_ is the DC conductivity, *x* the volume fraction of the conductive phase, *x_c_* the critical concentration and *t* the critical exponent.

Critical concentration was found to be 0.77% (w/w) of MWCNTs and critical exponent 1.54. 

All lignin-polyurethane compositions with MWCNTs’ concentration above the percolation threshold have sufficiently high conductivity for electrochemical sensor applications. The percolation threshold for composites with high aspect ratio fillers, such as CNTs, can be calculated using 3D statistical percolation model proposed in a previous study [19]. Nanotubes used in this work had a length of 1–5 µm and a diameter of 5–19 nm, which means that their aspect ratio could vary between 52 and 260 and predicted critical concentration between 0.18% and 1.46%. It is important to point out that statistical model does not account for interactions between filler and polymer matrix, effect of the processing conditions or curved shape of nanotubes. Nevertheless, experimental value of the critical concentration for LignoBoost^®^ kraft lignin-based polyurethane is within the predicted range. Critical concentrations between 0.5 to 4.5% (w/w) of CNTs were reported in the literature for the thermoplastic polymers [20]. Critical concentration obtained for LignoBoost^®^ kraft lignin-based polyurethane is higher compared to the value of 0.18% (w/w) previously reported for the conventional technical kraft lignin doped with MWCNTs with the same characteristics as the ones used in this work [16]. This disparity can be related to the differences in the two lignins’ structure and, consequently, the difference in the interaction between nanotubes and polymer matrix.

The LignoBoost^®^ kraft lignin possesses higher molecular weight than the conventional kraft lignin and higher content of phenolic groups [18], two factors favoring the association of lignin molecules in solution with copolymer (PPGDI). This negatively affects the dispersion of lignin in the polymeric matrix and, therefore, the dispersion of associated nanotubes in bulk. As such, the expected threshold occurred at a higher concentration of CNTs in the composite than that observed with conventional lignin kraft. According to previous findings, lignin interacts with CNTs via π-π stacking between aromatic ring and carbon nanotubes side wall, leading to the increase of the electron delocalization and eventual change in lignin chain conformation, thus allowing better π—overlap along the chain of lignin and giving rise to increased electric conductivity [16]. Oriented by lignin, CNTs interact with each other, forming conductive assembles, which are incorporated into the polymeric matrix. 

The critical exponent reflects the dimensionality of the system and usually takes values between 1.3 and 1.9, corresponding to two- and three-dimensional percolating systems, respectively [21,22]. Calculated value of the critical exponent for the doped LignoBoost^®^ kraft lignin-based polyurethane was lower than theoretical values, which is often observed for CNTs/polymer composites [20]. Such apparent reduction of system dimensionality was interpreted as a consequence of mutual attraction and realignment of carbon nanotubes during polymer curing. Strong nanotube–nanotube, lignin–nanotube, and nanotube–polymer matrix interactions are a prerequisite for such realignment to take place. Thus, the formation of the conducting network in the polymer is not a true statistical percolation process based on random distribution.

**Figure 2:**
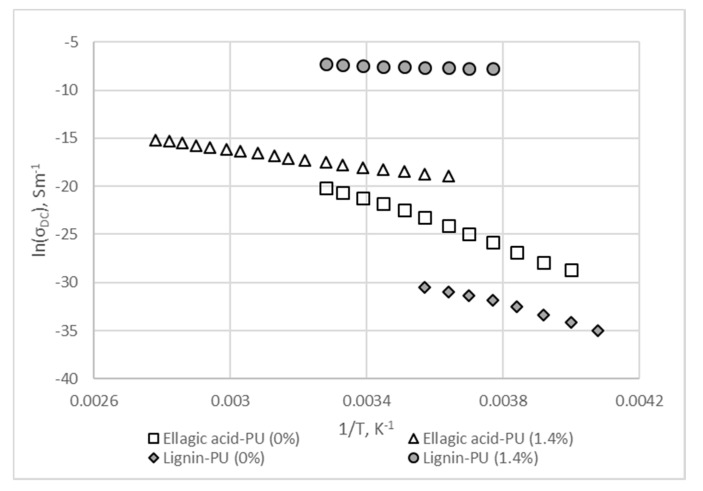
Logarithm of DC conductivity (σ_DC_) versus the inverse of the temperature for LignoBoost^®^ kraft lignin and ellagic acid based polyurethane polymer composites.

A DC conductivity of both lignin and ellagic acid-based polymers increases exponentially with temperature, as shown in the Figure 2. This behavior, which is typical for polymer composites, indicates that the conductivity is a thermally activated process and can be described by the well-known Arrhenius relation as follows

(2) $$\sigma_{DC}\propto exp[-\frac{E_{a}}{kT}]$$

where *E_a_* is the activation energy (J mol^−1^ K^−1^), *T* is the absolute temperature (K) and *k* is the Boltzmann constant, 1.380649 × 10^−23^ J·K^−1^ [23].

Activation energy can be calculated using plots of lnσ_DC_. vs. the inversed temperature (Figure 2). Values of the activation energy for the undoped LignoBoost^®^ kraft lignin and ellagic acid-based polymers were 0.76 eV and 1.05 eV, respectively. After addition of 1.4%(w/w) of MWCNTs, activation energy decreased to 0.08 eV and 0.39 eV for LignoBoost^®^ kraft lignin and ellagic acid-based polymers, respectively. Accordingly, the addition of MWCNTs resulted in a significant decrease of activation energy and increase of DC conductivity in both polymers, being more pronounced in lignin-based polyurethane, indicating active interaction between carbon nanotubes and lignin.

Ellagic acid-based polyurethanes had low conductivity even after doping with 1.4% (w/w) of MWCNTs, indicating that either no percolation occurs in this material or it occurs at relatively high concentrations of carbon nanotubes, making synthesis of such polymer costly and unpractical. Moreover, higher concentrations of a filler could alter thermoplastic properties of the polymer, which would be undesirable. Though both ellagic acid and lignin have aromatic groups in their structure, that were suggested to be involved in the interaction with carbon nanotubes, in the case of ellagic acid this interaction is certainly insufficient to effectively realign carbon nanotubes. This can be related to the significantly smaller size of the ellagic acid molecule compared to lignin, which prevents effective re-orientation of the carbon nanotubes. Hence, ellagic acid itself does not provide enough performance to be used in conducting blends with MWCNTs. Thus, only LignoBoost^®^ lignin-based polyurethanes were considered a perspective sensing material and studied in more detail. 

**Figure 3:**
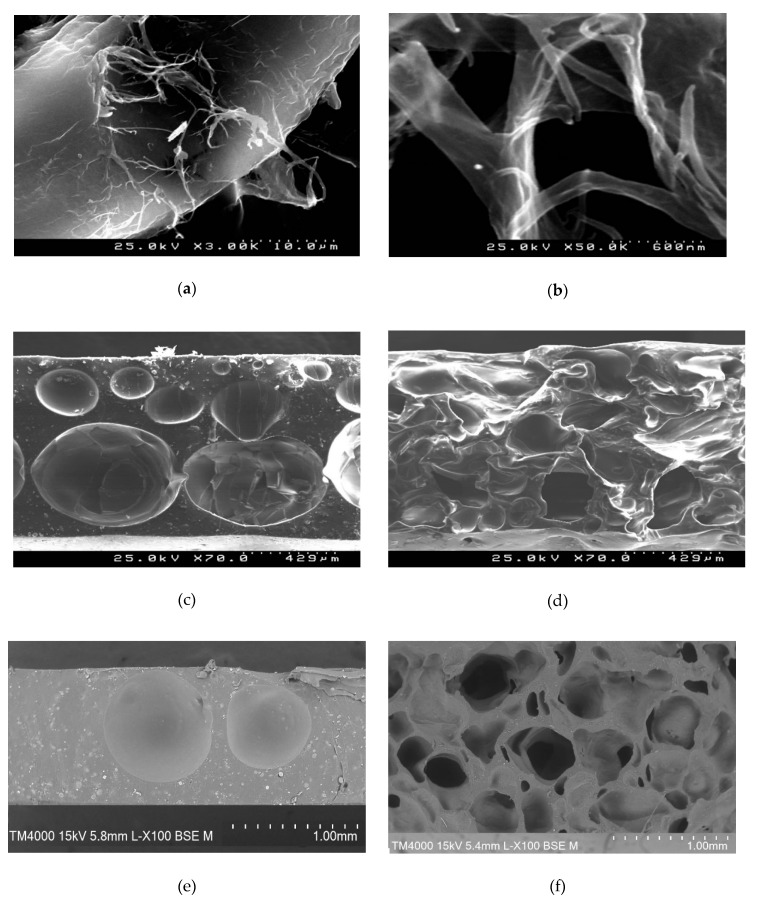
SEM images of: (**a**) and (**b**) LignoBoost^®^ kraft lignin mixture with MWCNTs (1%); (**c**) undoped LignoBoost^®^ kraft lignin-based polyurethane; (**d**) lignin-based polyurethane doped with 1.4% (w/w) of MWCNTs; (**e**) undoped ellagic acid-based polyurethane; (**f**) ellagic acid-based polyurethane doped with 1.4% (w/w) of MWCNTs.

Interaction between LignoBoost^®^ kraft lignin and MWCNTs was assessed by probing the effectiveness of the nanotube dispersion in lignin-based polyurethane. SEM images of the mixture of LignoBoost^®^ kraft lignin with MWCNTs (1.4% w/w) show an even distribution of disentangled carbon nanotubes, appearing as thin fibers on the surface of the larger lignin particles (Figure 3a,b). SEM images of the transversal cuts of LignoBoost^®^ kraft lignin-based polyurethane and ellagic acid-based polyurethane films, with and without MWCNTs, all differed in their structure. Both undoped polymers are porous, however, while lignin-based polyurethane has several evenly distributed air bubbles of varying sizes (Figure 3c), ellagic acid-based polyurethane is denser with fewer air bubbles in its structure (Figure 3e). Addition of the MWCNTs results in appearance of irregularly shaped pores in lignin-based polyurethane (Figure 3d) and numerous round shaped pores in ellagic acid-based polyurethane (Figure 3f). The structural dissimilarities between undoped and doped polymers confirm interaction between carbon nanotubes and lignin or ellagic acid. Differences in properties of ellagic acid and lignin, the size of the molecule probably being the most important, results in different interaction between these compounds and carbon nanotubes, thus leading to different structure of the respective polyurethanes. Further insights into the interactions between LignoBoost® lignin and carbon nanotubes in the polyurethane matrix were gained using dielectric spectroscopy.

### 3.2. AC Conductivity

**Figure 4:**
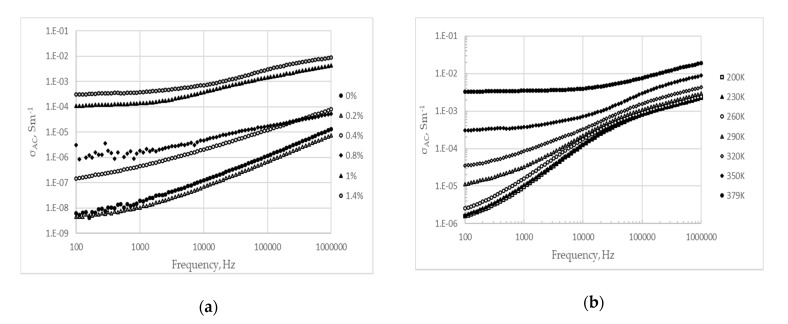
Frequency dependence of AC conductivity (σ_AC_) for (**a**) lignin-based polyurethane doped with different amounts of MWCNTs at T = 350 K; (**b**) lignin based polyurethane doped with 1.4% (w/w) of MWCNTs at different temperatures.

**Figure 5:**
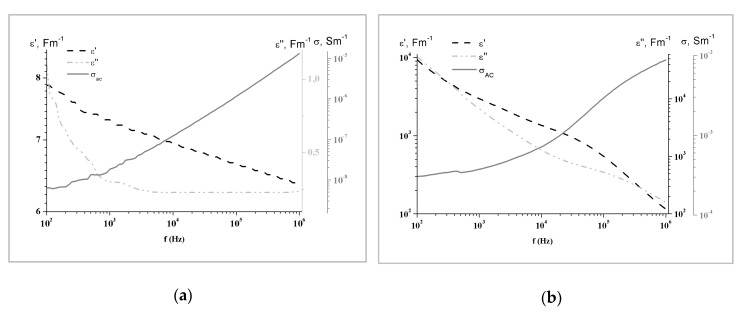
Real and imaginary parts of the complex permittivity, ε*(f) = ε′(f) − iε″(f), and the calculated electrical conductivity, σ*_AC_*, as function of frequency at T = 350 K for LignoBoost^®^ kraft lignin-based copolymer (**a**) undoped and (**b**) doped with 1.4% (w/w) MWCNTs.

Alternating current (AC) conductivity of LignoBoost^®^ kraft lignin-based copolymers undoped and doped with different amounts of MWCNTs is depicted in the Figure 4a. The effect of the temperature on the AC conductivity variation as a function of frequency for LignoBoost^®^ kraft lignin based polyurethane, doped with 1.4% (w/w) of MWCNTs, is shown in the Figure 4b. Real and imaginary parts of the complex permittivity, ε*(f) = ε′(f) − iε″(f), and calculated AC electrical conductivity as a function of frequency for the lignin-based copolymer, with 1.4% (w/w) and without MWCNTs at T = 350 K, are shown in the Figure 5a,b, respectively.

Increase of AC conductivity and decrease of both, real and imaginary parts of permittivity, with an increase of frequency was observed for both polymers (Figure 5a,b). Two distinct domains can be identified in the AC conductivity dependence on frequency [24]. AC conductivity is nearly independent of the frequency at low frequencies (below 1000 Hz), with its value approaching the DC conductivity (Figure 5a,b). This phenomenon, called the anomalous low frequency dispersion, is commonly observed in the disordered low-dimensional materials such as polymer matrices. Anomalous low-frequency dispersion arises from the restricted effective charge transport when charge motions are limited to one- or two-dimensional pathways. At frequencies above the crossover frequency fc, AC conductivity increases to different levels, depending on the material. Both ε’ and ε´´ decrease in all measured frequency intervals, which also corresponds to anomalous frequency dispersion.

Substantial increase of critical frequency, at which an increase in conductivity occurs, was observed in polymers doped with 0.8% (w/w) and higher concentrations of MWCNTs (Figure 4a). The critical frequency increased concomitantly with MWCNTs’ concentration in the polymer, which is common behavior for the polymers doped with conducting particles [25,26]. The effect of the temperature on the AC conductivity dependence of the frequency for lignin based polyurethane doped with 1.4% (w/w) of MWCNTs is shown in the Figure 4b. Concomitantly dependent on applied frequency, with only a small increase observed at high frequencies. At lower temperatures, the increase in conductivity is more pronounced as the frequency increases.

### 3.3. Polymer Characterization

Apart from electrical conductivity, other LignoBoost^®^ kraft lignin-based polymer properties were not significantly altered by doping with MWCNTs. No differences were observed in FT-MIR spectra of doped and non-doped polymers (Figure S1). Doping with carbon nanotubes also did not affect glass transition temperature of LignoBoost^®^ kraft lignin-based polyurethanes, which was found to be −50 °C and −49 °C, with and without MWCNTs doping, respectively (Figure S2a,b). Low glass transition temperature is associated with the prevalence of flexible segments of the PPGDI chains, which is the major component of the polymer, and the possible disintegration of lignin molecules. It is worth noting that low glass transition temperatures of a polymer are required for its use as self-plasticizing sensing membrane [27].

Incorporation of the Lignoboost^®^ kraft lignin into the polymeric matrix improved its thermal stability as evidenced by the increase of the maximum degradation temperature from 355 °C for lignin to 388 °C for the lignin-based polyurethane (Figure S3a,b, respectively). Increase of the thermal stability of polyurethane is related to the consumption of the hydroxyl groups of lignin in the polymerization reaction with isocyanate groups, and thus, to the decrease of the amount of functional groups susceptible to the thermal degradation [14]. MWCNTs did not significantly change the thermal behavior of lignin-based polyurethane, with maximum degradation temperature of doped polymer and undoped polymers being 387 °C and 388 °C, respectively (Figure S3b). This finding is in agreement with literature data and can be explained by the more heterogeneous structure of doped polyurethane, which was also observed by SEM, resulting from the interaction of MWCNTs with lignin hydroxyl groups, which makes the latter inaccessible for the reaction with isocyanate.

### 3.4. Sensor Properties

**Figure 6:**
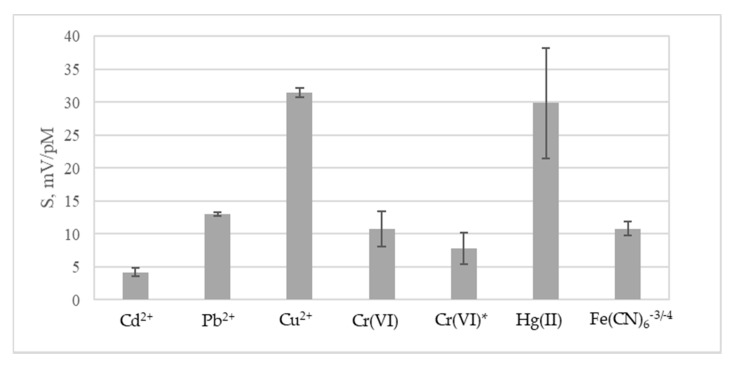
Slopes of the electrode function of LignoBoost^®^ kraft lignin-based sensor. Mean values of three calibrations with standard deviations are shown.

LignoBoost^®^ kraft lignin-based polyurethane doped with 1.4% (w/w) of MWCNTs was used for the preparation of the membrane of potentiometric chemical sensor. Slopes of the electrode function (S) of the sensor in the individual solutions of transition metal salts are depicted in the Figure 6. The sensor displayed no response to sodium, calcium and iron (III), very low response to cadmium, lead, and chromium (VI), and low redox response in the solutions of redox pair Fe(CN)_6_/Fe(CN)_6_^−3/−4^. Sensor displayed theoretical response of 32 (±1) mV·pM^−1^ to copper (II) with detection limit of 6(±1) × 10^−6^ mol·L^−1^. Sensor response to mercury (II) decreased with each consecutive calibration from 32 mV·pM^−1^ in the first calibration to 18 mV·pM^−1^ in the third. After exposure to the solutions of mercury (II), the sensor no longer responded to copper (II) ions. This behavior may be associated with irreversible complexation of mercury (II) ions by the polymeric membrane similar to the reported for several copper-sensitive organic ionophores [28]. Thus, mercury must be avoided in the solutions analyzed by the sensor. Sensor displayed high selectivity towards copper in the presence of other transition metals except for mercury (II), to which it was not selective (Table 1). Parameters of the sensor are close to the values reported in the literature for the copper-selective electrodes based on organic ligands (Table S1). 

Sensing characteristics of the sensor with LignoBoost^®^ kraft lignin-based membrane differs from the ones with membranes prepared using other technical lignins, such as conventional kraft lignin precipitated by deep acidification of black liquor, lignosulfonate, and organosolv lignin, all of which responded selectively to Cr(VI) in acidic solutions [16]. Mechanism of these sensors’ response to Cr(VI) was suggested to be mixed, redox, and ionic, as the sensitivity of sensors was correlated with the content of quinone structures, the dominant redox-active moieties of lignin [29]. Contrary to these findings, LignoBoost^®^ kraft lignin-based sensor did not display redox sensitivity, showing a very low response to Cr(VI) and redox pair, but instead it showed a selective response to Cu(II). 

This behavior can be explained by the differences in the composition of eucalyptus LignoBoost^®^ kraft lignin and other technical lignins [30,31,32], most noticeably, lower content of redox quinone type moieties and significantly higher content of polyphenolic groups with vicinal hydroxyls originating from concomitant tannins in the former [18]. In particular, the LignoBoost^®^ kraft lignin has a higher total content of total hydroxyl groups and higher relative content of phenolic hydroxyl groups compared to the technical kraft lignin obtained from the cooking of the same wood species, but isolated by the conventional procedure [32]. Considering that sensors based on the polyurethanes, synthesized using kraft lignin isolated using conventional procedure, did not display sensitivity to Cu(II) [16], response of the sensor developed in this work can be attributed to the capability of phenolic hydroxyl groups to complex transition metals with higher specificity towards copper and mercury. This proposition is further corroborated by the reported higher chelating capacity of tannins with vicinal phenolic groups towards Cu(II) when compared to other bivalent transition ions, such as Zn(II) and Fe(II) [33]. However, studies on the exact mechanisms are still necessary for a better understanding of the observed phenomena.

**Figure 7:**
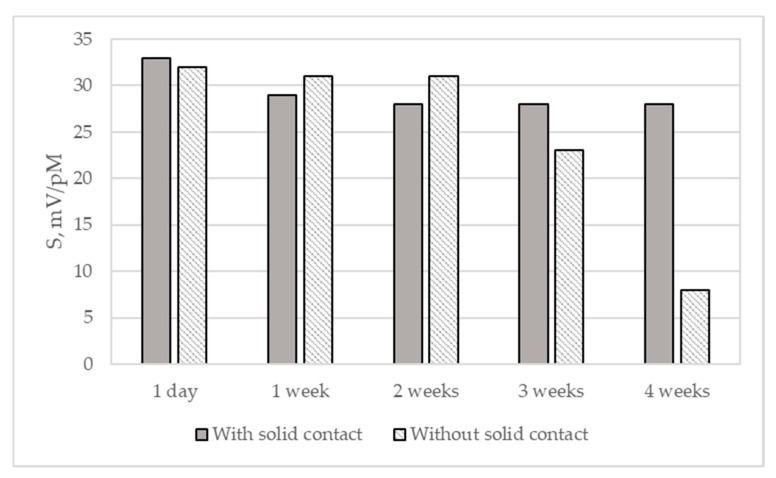
Slopes of the electrode function of the LignoBoost^®^ kraft based sensors with and without solid contact in Cu(II) solutions during four weeks.

Furthermore, characteristics of sensors with and without solid inner contact, as well as their stability in copper(II) solutions over a 4-week period were evaluated. Potentiometric chemical sensors require inner contact since ion-to-electron transduction between the sensing membrane with ionic conductivity and substrate with electronic conductivity ensures the stability of the sensor potential. In all-solid-state sensors, inner contact is commonly made from conducting polymers that have mixed ion-electronic conductivity [1]. As lignin-based polyurethanes are conducting polymers with mixed ionic and electronic conductivity, it was expected that they could be used for fabrication of the all solid-state sensors without additional inner contact layer. Newly prepared sensors with lignin-based polyurethane membrane, both with and without solid inner contact, displayed responses close to the theoretical ones to copper (II) ions: 33 and 32 mV·pM^−1^, respectively, being not statistically different for *p* = 0.05 (Figure 7). However, sensor without solid inner contact had higher detection limit than the one with solid inner contact: 2(±1) × 10^−5^ and 6(±1) × 10^−6^ mol·L^−1^, respectively. After one week, the slope of the sensor with solid inner contact slightly decreased, from 33 to 29 mV·pM^−1^ and remained constant over the next three weeks (slopes were not statistically different for *p* = 0.05). The slope of the sensor without solid inner contact remained close to the theoretical value for two weeks, after which it started to decrease. After the fourth week, this decrease became abrupt, with sensor’s response to Cu(II) being only 8 mV·pM^−1^ (statistically different for *p* = 0.05). A slight increase of the detection limit was observed for both types of sensors during this period.

Deterioration of properties, such as long-term stability and detection limit, of the sensor without solid inner contact indicates that the interface between electrode and membrane is not well-defined [34]. This may occur due to the poor adhesion of the lignin-based polyurethane to the gold working electrode, leading to the formation of the water layer. Therefore, despite the electronic conductivity of lignin-based polyurethane imparted by MWCNTs, an intermediate conductive polymer layer is required to ensure better temporal stability and a lower detection limit.

## 4. Conclusions

Conducting composite polyurethane, based on LignoBoost^®^ kraft lignin and doped with MWCNTs, was demonstrated to be a promising material for sensing applications. Electrical conductivity and impedance spectroscopy measurements revealed that the interaction between carbon nanotubes and lignin molecules in the polymer enhances its electrical conductivity. The percolation threshold of 0.77% (w/w) of MWCNTs was observed in LignoBoost^®^ kraft lignin-based polyurethane but not in ellagic acid-based one, highlighting the importance of the presence of conjugated aromatic structure for the percolation effect to occur. LignoBoost^®^ kraft lignin-based polymer doped with MWCNTs was used to manufacture all solid state potentiometric chemical sensors, which displayed high sensitivity and selectivity to Cu(II) and long-term stability. The dissimilarity between sensing properties of developed sensors and potentiometric sensors based on technical lignins was assigned to the higher content of polyphenolic groups originating from tannins, namely the vicinal phenolic hydroxyl groups in LignoBoost^®^ kraft lignin and lower content of quinone type moieties, which results in enhanced ion-exchanged properties. 

## Figures and Tables

**Table 1:** Logarithm of the selectivity coefficients, logK_ij_, of LignoBoost^®^ kraft based sensor to copper(II) determined using mixed solution method. Mean values of four measurements with standard deviations are shown.

Interferent	Cd(II)	Pb(II)	Cr(VI)	Hg(II)
logK_ij_	−1.68 (±0.1)	−1.49 (±0.05)	−1.6 (±0.2)	0.2 (±0.1)

## Supplementary Materials

The following supplementary data are available online at https://www.mdpi.com/1996-1944/13/7/1637/s1, Figure S1: FT-MIR spectra of LignoBoost^®^ kraft lignin-based polymers undoped and doped with 1.4% (w/w) MWCNTs; Figure S2a,b: DSC curves of LignoBoost^®^ kraft lignin-based polyurethane undoped (a) and doped with 1.4% (w/w) MWCNTs (b); Figure S3a,b: TGA curves of LignoBoost^®^ kraft lignin (a) and LignoBoost^®^ kraft lignin-based polyurethane undoped and doped with 1.4% (w/w) MWCNTs (b); Table S1: Comparison of the performance characteristics of some copper ion sensors based on organic ionophores reported in the literature and developed in this work.

## Abbreviations

AC conductivity	Alternating current conductivity
DC conductivity	Direct current conductivity
DSC	Differential scanning calorimetry
FT-MIR	Fourier transform–mid-infrared spectroscopy
MWCNT	Multiwall carbon nanotubes
PPGDI	Poly(propylene glycol), tolylene 2,4-diisocyanate terminated
SEM	Scanning electron microscopy
TGA	Thermogravimetric analysis
