# Supplementary material for: Nanocomposite Polymeric Materials Based on Eucalyptus Lignoboost® Kraft Lignin for Liquid Sensing Applications
Source: Materials (Basel). 2020 Apr 2;13(7):1637. doi: 10.3390/ma13071637 (PMC7178355; doi:10.3390/ma13071637)
Supplement: Supplementary file 1 [file materials-13-01637-s001.pdf]

materials

Supplementary Materials

Nanocomposite Polymeric Materials Based on Eucalyptus Lignoboost® Kraft Lignin for Liquid Sensing Applications

Sónia S. Leça Gonçalves [1], Alisa Rudnitskaya [2,*], António J.M. Sales [3], Luís M. Cadillon Costa [3], and Dmitry V. Evtuguin [1,*]

1 CICECO and Department of Chemistry, University of Aveiro, Aveiro, 3810-193, Portugal; leca.sofia@ua.pt
2 CESAM and Department of Chemistry, University of Aveiro, Aveiro, 3810-193, Portugal
3 I3N and Department of Physics, University of Aveiro, Aveiro, 3810-193, Portugal; jsales@ua.pt (A.J.M.S.); kady@ua.pt (L.C.C.)
* Correspondence: alisa@ua.pt (A.R.); dmitrye@ua.pt (D.V.E.)

Received: 16 February 2020; Accepted: 30 March 2020; Published: date

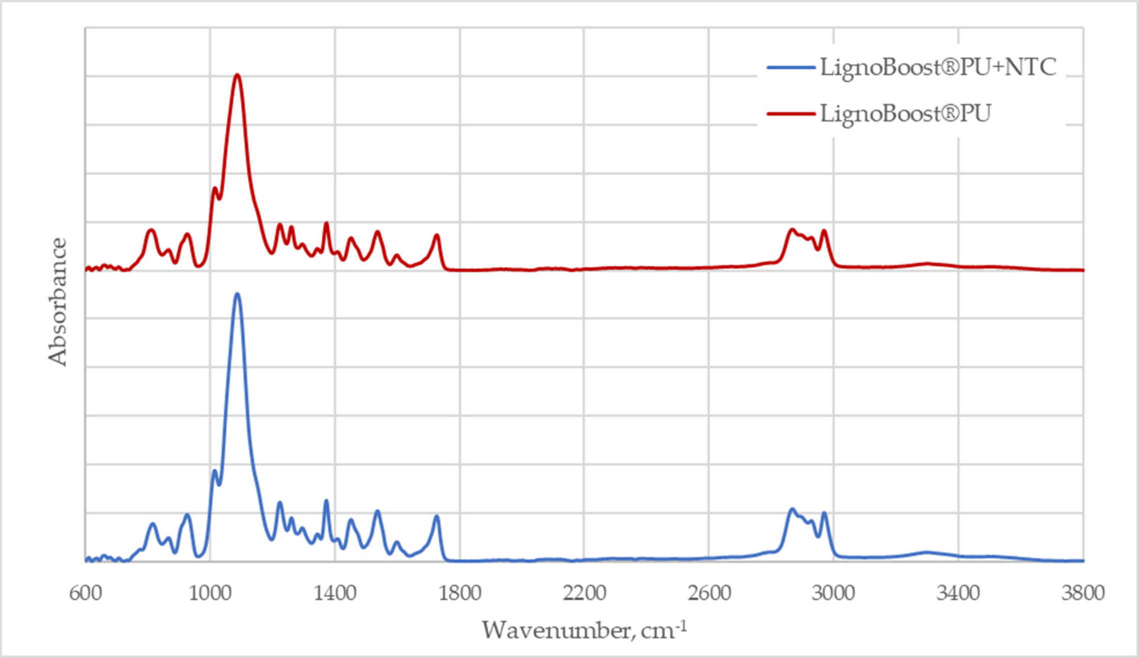

Wavenumber, cm^{-1}	Band assignment
2970, 2916 and 2868	CH stretching (symmetric and asymmetric) in CH_3 and CH_2
1733	C=O stretching (urethane group)
1536	NH bend (secondary amine in urethane group)
1372	O-CO stretching (urethane group)
1090	C-O vibration (polyether bridges)

Figure S1. FT-MIR spectra of LignoBoost® kraft lignin-based polymers undoped (LignoBoost®PU) and doped with 1.4% (w/w) MWCNTs(LignoBoost®PU + MWNTC).

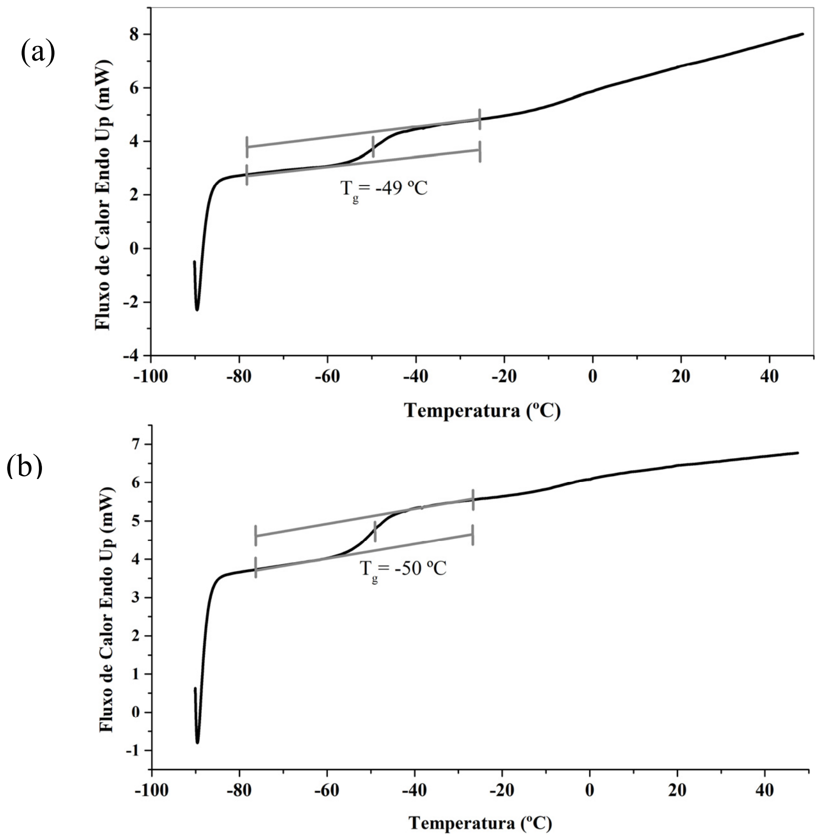

Figure S2. DSC curves of LignoBoost® kraft lignin-based polyurethane undoped (**a**) and doped with 1.4% (w/w) MWCNTs (**b**).

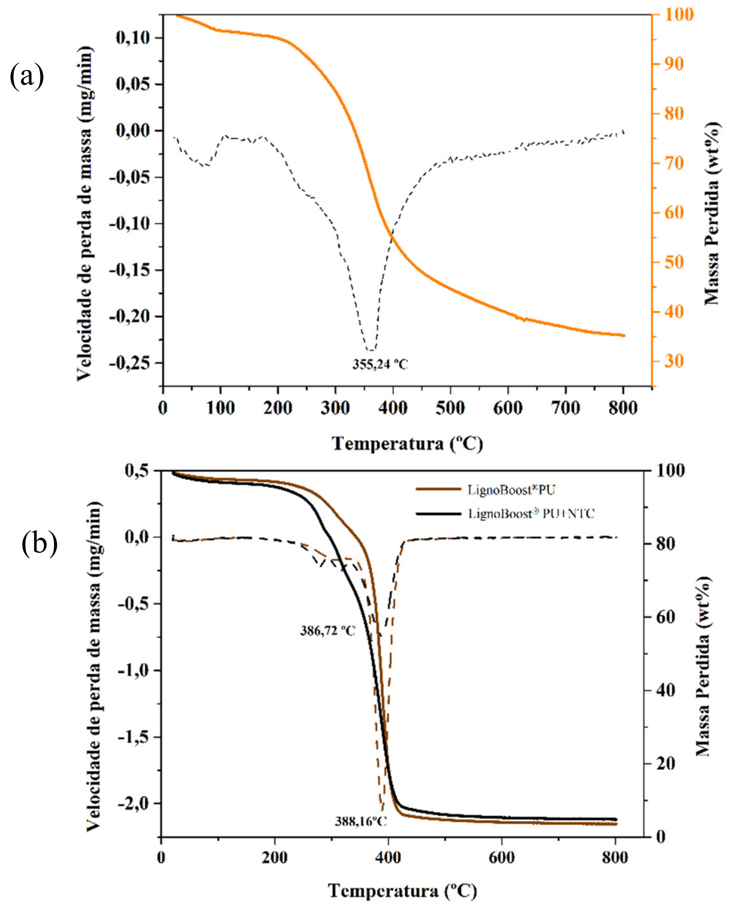

Figure S3. TGA curves of LignoBoost® kraft lignin (**a**) and LignoBoost® kraft lignin-based polyurethane undoped and doped with 1.4% (w/w) MWCNTs (**b**).

Table S1. Comparison of the performance characteristics of some copper ion sensors based on organic ionophores reported in the literature and developed in this work.

Slope, mV/pCu	Detection Limit, mol·L^{-1}	Linear Range, mol·L^{-1}	Ref.
28.7	1×10^{-6}	1.0×10^{-6}–1.0×10^{-2}	1
28.3	8.3×10^{-7}	1.0×10^{-6}–1.0×10^{-1}	2
28.8	6.3×10^{-7}	2×10^{-6}–5×10^{-3}	3
34.2	7×10^{-6}	8×10^{-6}–1.0×10^{-2}	4
29.3	4×10^{-6}	4.4×10^{-6}–1.0×10^{-1}	5
32	6×10^{-6}	8×10^{-6}–1.0×10^{-3}	This work

References

1. Faridbod, F.; Davarkhah, N.; Beikzadeh, M.; Yekefallah, M.; Rezapour, M. Cu^{2+} -selective Sensors Based on a New Ion-Carrier and Their Application for the Analysis of Copper Content of Water Samples. *Int. J. Electrochem. Sci.* **2017**, *12*, 876–889, doi:10.20964/2017.02.11.
2. Andac, M.; Coldur, F.; Bilir, S.; Birinci, A.; Demir, S.; Uzun, H. View Solid-contact polyvinyl chloride membrane electrode based on the bis[(2-(hydroxyethylimino)phenolato]copper(II) complex for trace level determination of copper ions in wastewater. *Can. J. Chem.* **2014**, *92*, 324–328, doi:10.1139/cjc-2013-0530.
3. Kopylovich, M.N.; Mahmudov, K.T.; Pombeiro, A.J.L. Poly(Vinyl) Chloride Membrane Copper-Selective Electrode Based on 1-Phenyl-2-(2-Hydroxyphenylhydrazo)Butane-1,3-Dione. *J. Hazard. Mater.* **2011**, *186*, 1154–1162, doi:10.1016/j.jhazmat.2010.11.119.
4. Kamel, A.H.; Mahmoud, W.H.; Mostafa, M.S. Response Characteristics Of Copper-Selective Polymer Membrane Electrodes Based On A Newly Synthesized Macrocyclic Calix[4]Arene Derivative As A Neutral Carrier Ionophore. *Electroanalysis* **2010**, *22*, 2453–2459, doi:10.1002/elan.201000187.
5. Gupta, V.K.; Jain, A.K.; Maheshwari, G.; Lang, H.; Ishtaiwi, Z. Copper(II)-Selective Potentiometric Sensors Based On Porphyrins In PVC Matrix. *Sens. Actuators B Chem.* **2006**, *117*, 99–106, doi:10.1016/j.snb.2005.11.003.

© 2020 by the authors. Submitted for possible open access publication under the terms and conditions of the Creative Commons Attribution (CC BY) license (http://creativecommons.org/licenses/by/4.0/).
